# β-blocker Therapy in Cholinergic Urticaria, Adrenergic Urticaria and the Aquagenic Continuum Beta-Blocker Therapy in Chronic Inducible Urticaria

**DOI:** 10.1007/s12016-026-09175-1

**Published:** 2026-07-02

**Authors:** Evelien M. Hutten, Rick G. Pleijhuis

**Affiliations:** 1 Department of Internal Medicine and Allergology, University Medical Center Groningen, AA21. Hanzeplein 1, Groningen, 9713 GZ the Netherlands; 2 Department of Allergology, Frisius Medical Center, Heerenveen, The Netherlands

**Keywords:** Adrenergic Beta-Antagonists (D000319), Chronic Inducible Urticaria (D000094482), Chronic Urticaria (D000080223), Urticaria (D014581), Quality of Life (D011788)

## Abstract

Chronic urticaria is a common skin disorder that substantially affects both daily functioning and quality of life. Among its subtypes, chronic inducible urticaria often presents with greater therapeutic challenges than chronic spontaneous urticaria. A comprehensive review of the available evidence indicates that β-blockers may offer a rapid-acting, well-tolerated, and effective treatment option for patients with specific subtypes of chronic inducible urticaria specifically cholinergic urticaria, adrenergic urticaria, and the aquagenic continuum. This approach introduces a readily available, non-parenteral, and cost-efficient therapeutic alternative that could also yield novel insights into the underlying pathophysiology of urticaria. In addition, this manuscript underscores the need to update current clinical guidelines by: (1) recognizing adrenergic urticaria as a distinct chronic inducible urticaria subtype; (2) broadening the conceptual framework for aquagenic-induced symptoms; and (3) standardizing intradermal test concentrations for adrenergic and cholinergic urticaria.

## The Clinical Problem

Chronic urticaria is a common skin disorder, often associated with severe pruritus, that impairs both objective functioning and subjective well-being [1–5]. It affects a considerable part of the global population, with a lifetime prevalence of 1.4% [6]. Chronic urticaria can be classified as chronic spontaneous urticaria, chronic inducible urticaria, or a combination of both. The spontaneous form occurs without any specific triggers, whereas the inducible form can be consistently elicited by reproducible stimuli and is further subclassified as symptomatic dermographism, cold urticaria, delayed pressure urticaria, solar urticaria, heat urticaria, vibratory angioedema, cholinergic urticaria, contact urticaria, and aquagenic urticaria in the latest guideline [7]. Patients may simultaneously present multiple forms of chronic urticaria including more than one chronic inducible urticaria subtype.

Chronic inducible urticaria is generally considered more difficult to treat than chronic spontaneous urticaria, although available studies for this subtype are limited by sample sizes, heterogeneous efficacy outcomes, and poor reporting quality [8–10]. While avoidance of relevant triggers can be effective, it is often difficult or even impossible for patients to achieve. First-line therapy with a second-generation antihistamine - up to four times the standard daily dose - frequently fails to induce complete remission [9]. The effectiveness of the recommended second-line treatment, off-label omalizumab, is likewise often limited in chronic inducible urticaria patients [7, 11]. A variety of new drugs are currently under investigation in this patient group, including targeted monoclonal antibodies, JAK-inhibitors, KIT-inhibitors, and Bruton’s tyrosine kinase inhibitors [10, 12, 13]. Nonetheless, access to these new treatments may prove particularly challenging in low- and middle-income countries because of limited availability and affordability. As such, these difficult-to-treat chronic urticaria endotypes still remain an important unmet need.

Interestingly, based on our experience and a thorough review of the literature, β-blockers could serve as a fast-acting, well-tolerated, and effective treatment option in specific, less common subtypes of chronic inducible urticaria, namely cholinergic urticaria, adrenergic urticaria, and the aquagenic disease continuum. This would introduce a widely available, non-parenteral, and relatively inexpensive treatment option for these specific subgroups.

## Background

### Cholinergic urticaria and Adrenergic urticaria

Both cholinergic urticaria and adrenergic urticaria can be evoked by physical and/or emotional stress. Adrenergic urticaria, as a subtype of chronic inducible urticaria, is reported less frequently than cholinergic urticaria; although its prevalence may be underestimated, as is not included in the latest classification systems [7]. Cholinergic urticaria is characterized by pruritus, erythema, and pronounced papular whealing, triggered by exercise, passive warming, emotional stress, and/or consumption of spicy or hot food. Adrenergic urticaria might be mistaken for cholinergic urticaria due to overlapping triggers. However, in adrenergic urticaria, the erythematous papules are surrounded by white halos of blanched, vasoconstricted skin, whereas in cholinergic urticaria, the small papules are surrounded by large, erythematous flares [14]. Both cholinergic urticaria and adrenergic urticaria must be distinguished from exercise-induced anaphylaxis, in which anaphylaxis is triggered by physical activity but not by passive heating. Within the group of patients with exercise-induced anaphylaxis, one subgroup develops reactions provoked exclusively by exercise, whereas another experiences episodes solely when exercise follows prior ingestion of a specific food [15].

### Aquagenic Urticaria, Aquagenic Pruritus and Aquadynia: the Aquagenic Induced disease Continuum

Aquagenic urticaria, a subtype of chronic inducible urticaria, is reported as very rare [16]. In this form of chronic inducible urticaria, symptoms develop when the skin is exposed to water, including sweat and tears, regardless of temperature. This has a profound impact on quality of life, as trigger avoidance is virtually impossible. In the international classification, neither aquagenic pruritus nor aquadynia are mentioned as part of a clinical continuum of aquagenic-induced skin phenomena [7]. Similarly, most authors make a strict distinction between aquagenic urticaria and aquagenic pruritus without visible skin manifestations, as the latter may be associated with systemic diseases such as polycythemia vera or other myelodysplastic syndromes [17]. The absence of visible symptoms, however, is not pathognomonic for an underlying hematological disease [18, 19]. We recently hypothesized that aquagenic pruritus, aquadynia (with or without rash or plaques), and aquagenic urticaria represent a disease continuum [20].

### Use of β-blockers in Specific Chronic Inducible Urticaria Subtypes

An important unmet need in aforementioned chronic inducible urticaria subtypes is the existence of difficult-to-treat endotypes not responsive to antihistamines. As per the literature, multiple cases of adrenergic urticaria (*n* = 21) [14, 21–32], adrenergic pruritus (*n* = 1) [24], cholinergic urticaria (*n* = 5) [33–35], the aquagenic spectrum (*n* = 11) [18–20, 36–38], and combinations of multiple subtypes of chronic inducible urticaria (*n* = 3) [39–41] show beneficial results of treatment with β-blockers (Tables 1 and 2). In most cases, the treatment demonstrated both impressive efficacy and good tolerability, even in patients for whom antihistamines had previously failed. To our knowledge, no randomized controlled trials have evaluated this treatment. Several reports describing favorable responses to β-blockers also note that symptoms recurred following discontinuation of therapy and resolved when therapy was resumed. The non-selective β-blocker propranolol was used most frequently. In three cases, selective β-1 adrenergic blockers (atenolol and bisoprolol) were prescribed [14, 18, 22]; however, in two of them these drugs proved ineffective. In these cases, subsequent treatment with propranolol was successful. Interestingly, case reports indicate that the required β-blocker dose to prevent urticaria varies widely, ranging from 10 mg on-demand to 240 mg propranolol daily, with lower doses generally sufficient for aquagenic-induced symptoms compared to cholinergic urticaria. The lack of response to propranolol in one case report of adrenergic urticaria may have been related to an inadequate dosage since as all reported effective doses in this chronic inducible urticaria subtype were higher (cumulative doses of 60 mg – 120 mg per day) than the administered 20 mg once a day in this case (Table 1) [42]. Knowledge gaps regarding the optimal dose of propranolol are present, even for indications other than chronic inducible urticaria. It remains unclear to what extent the starting dose should be based on body weight, body mass index, or ideal body weight [43]. Most studies on propranolol in inducible urticaria do not report these parameters, making it difficult to assess whether they may explain differences in outcomes with similar doses in inducible urticaria on how drug dosing regimens can improve clinical outcomes. In another report, a cumulative dose of 300 mg short-acting propranolol resulted in treatment failure, whereas a once-daily dose of 160 mg long-acting propranolol resulted in near-complete remission [34]. The observed difference in treatment response may be explained by the more stable plasma concentrations and sustained β-adrenergic receptor blockade provided by long-acting formulas compared with short-acting preparations, which are associated with fluctuating drug levels and potential troughs below the therapeutic threshold.

**Table 1:** Characteristics of included case-series on usages of β-blockers in adrenergic and cholinergic induced urticaria and/or pruritus

Author, year	Type of study	Sex, Age	Diagnosis	Efficacy of earlier treatment attempts	Efficacy of β-blocking treatments
Ammann, 1999 [33]	Case report	M, 26	5-year history of cholinergic urticaria (IDT-P: ND)	No effect of histamine antagonists (both H1 and H2).	Propranolol 80 mg 2 times a day. At evaluation 1 month later: complete remission.
Capella, 2012 [21]	Case report	F, 45	History of adrenergic urticaria (IDT-P: A + ve)	ND	First responded to propranolol 20 mg 2 times a day. Later unresponsive to propranolol up to 40 mg 3 times a day, coupled with hydroxyzine, ranitidine, or even intramuscular chlorpheniramine). Meanwhile, rheumatoid arthritis became apparent. The courses of the diseases strictly paralleled each other.
Chedraoui, 2008 [22]	Case report	F, 64	1-year history of adrenergic urticaria. (IDT-P: AchH-ve, A + ve, NA + ve)	Responded promptly but partially to antihistamine therapy. Recurrence shortly upon discontinuation of antihistamine was the rule.	Not under control despite the intake of bisoprolol for hypertension. Upon substitution of bisoprolol with propranolol 20 mg 3 times a day, complete remission. Follow up for 4 months: symptom free, the rash recurred shortly after discontinuation of propranolol.
Conway, 1982 [34]	Case series	M, 26	7-year history of cholinergic urticaria (IDT-P: ND)	Anticholinergics, antihistamines and tranquillizers “without much success”.	Propranolol 80 mg 3 times a day: full control. Recurrence when missing a couple of doses. Follow up for 7 months.
		F, 24	3-year history of cholinergic urticaria and a non-specific rash in the two years before she had her first true attack (IDT-P: ND)	ND	Short acting propranolol not successful, even with 300 mg daily. Long-acting preparation of propranolol 1dd160mg gave almost complete relief.
		M, 22	2-year history of cholinergic urticaria, provoked only by his twice weekly game of squash (IDT-P: ND)	Probanthine 15 mg 1 h before squash: no effect.	Propranolol 40 mg before the game: no further attacks.
Feinberg, 2008 [35]	Case report	F, 22	2-year history of cholinergic urticaria (IDT-P: ND)	Loratadine, ranitidine, and oral steroids without any sustained success. Hydroxyzine: minimal improvement, but too sedating. Cetirizine 10 mg twice a day provided significant improvement, but incomplete resolution.	Montelukast, 10 mg once a day, and propranolol 20 mg twice a day, were added. Complete remission.
Goodman, 2021 [23]	Case report	M, 18	Adrenergic urticaria (IDT-P: ND)	Nonresponsive to high-dose second-generation H1-antihistamines, H2-antihistamines, montelukast, dapsone, and omalizumab monthly injections.	Good control after the combination of dupilumab monthly, propranolol 20 mg 2 times a day, and an antihistamine. Later, the patient had a flare-up of symptoms owing to hot weather and resumption of classes; after up titration of propranolol to 60 mg 2 times a day, symptoms resolved completely. A year later, dupilumab injections were temporarily paused owing to the pandemic, and his symptoms recurred. The patient is soon to be restarted on therapy.
Haustein, 1990 [24]	Case series	M, 51	2-year history of adrenergic urticaria (IDT-P: AM-ve, A + ve, NA + ve; IDT-C: A-ve, NA-ve)	Clemastine 2 mg and ketotifen 2 mg provided relief during attacks.	Propranolol 25 mg 3 times a day and tolazoline suppressed attacks. It reappeared after discontinuing therapy.
		F, 34	3-year history of adrenergic pruritus (IDT-P: itching reproduced by IDT with A and NA but not with AM)	ND	Propranolol 25 mg 2 times a day and tolazoline blocked itching.
Hogan, 2014 [25]	Case series (*N* = 2)	ND	Adrenergic urticaria		“Our experience with 2 patients with classic clinical lesions and complete response to propranolol”
Kawakami, 2015 [42]	Case report	M, 16	5-year history of adrenergic urticaria (IDT-P: AchH–ve, NA + ve)	Olopatadine (H-1 antagonist) and lafutidine (H-2 antagonist) failed to relieve symptoms. Oral clotiazepam 5 mg 3 times a day dramatically reduced the severity and frequency of the attacks and his subjective symptoms in a nervous state. The symptoms of chronic urticaria also gradually diminished with clotiazepam treatment, and he was satisfied with the effect of clotiazepam.	Propranolol 20 mg once a day had no effect on his symptoms. Carvedilol, an alpha- and β-blocker, reduced the urticarial lesions. However, carvedilol failed to relieve his subjective symptoms, such as tingling sensation and heart palpitations. He mainly had subjective symptoms; therefore, he was not satisfied with the effect of carvedilol.
Maerens-Tchokoka, 1999 [26]	Case report	M, 53	9-month history of adrenergic urticaria (IDT-P: ND)	ND	Propranolol 20 mg 3 times a day suppressed urticaria. The β-blocker was stopped and the urticaria recurred. Treatment was resumed and brought about complete control. Follow up for 21 months.
Mihara, 2008 [39]	Case report	F, 25	2-year history of cholinergic and adrenergic urticaria (IDT-P: AchH+ve, NA + ve)	Antihistamines and leukotriene antagonists failed to relieve symptoms.	Propranolol 10 mg 3 times a day was added to het existing drugs. Complete control. Follow up for 8 months.
Nishino, 2025 [40]	Case report	M, 26	8-year history of adrenergic urticaria complicated with acquired idiopathic generalized anhidrosis with cholinergic urticaria (IDT-P: AchH-ve, A + ve, NA + ve, DOS+ve)	No effect of H1-antihistamines and antileukotrienes.	Propranolol 30–60 mg per day: partial remission.
Ollaik, 2020 [27]	Case report	F, 39	4-month history of adrenergic urticaria (IDT-P: A + ve)	Unsuccessfully treatment with steroids, with per os steroids at a dose of 50 mg daily with slow tapering over a period of 3 weeks. No improvement with second generation of antihistamines.	Propranolol 40 mg 3 times a day: complete remission. Follow-up on 3 months showed controlled rash on propranolol.
Pesqué, 2023 [28]	Case report	M, 16	15-month history of adrenergic urticaria (IDT-P: A + ve)	Failed to respond to antihistamines at fourfold doses for 6 months and to omalizumab (300 mg/4 weeks) for 6 more months.	Propranolol up to 40 mg 3 times a day: partial improvement, but patient-reported outcome measures revealed poor control, important disease activity and a large effect on quality of life. Therapy was discontinued in favor of clotiazepam and associated with low doses of carvedilol (non-selective β-blocker and α-blocker activity). The intensity and frequency of the flares worsened significantly. Danazol was started, combined with carvedilol, and despite not being totally controlled, the patient improved with sustained response.
Schnebert [29]	Case report	M, 9	5-month history of adrenergic urticaria (IDT-P: A + ve; IDT-C (*n* = 5): A-ve)	H1-antihistamines up to 4 times the recommended dosage were not effective.	Propranolol progressively increased to 40 mg per day partially improved skin lesions.
Shelley, 1985 [14]	Case series	M, 28	1-year history of adrenergic urticaria (IDT-P: AchH–ve, A + ve, NA + ve; IDT-C (*n* = 7): AchH-ve, A–ve, NA-ve)	Hydroxyzine 50 mg, and diphenhydramine 50 mg provided relief during attacks.	Propranolol 20 mg 3 times a day prevented attacks. When propranolol was discontinued for 72 h, the hives reappeared. A trial of an alternative β-blocker, atenolol, was not successful. Follow up for 4 months.
		F, 40	17-year history of adrenergic urticaria (IDT-P: AchH–ve, A: only blanching but no lesion, NA +ve)	ND	Propranolol 10 mg 4 times a day decreased severity and frequency.
Silpa-archa [30]	Experience of authors		6 patients with adrenergic urticaria	ND	“Our six patients responded to propranolol”
Vithayasai, 1989 [31]	Case report	F, 22	10-year history of adrenergic urticaria (IDT-P: A + ve)	ND	Propranolol 20 mg and diazepam 2 mg prevented attacks.
Wang, 2013 [32]	Case report	F, 37	3-months-history of adrenergic urticaria (IDT-P: NA + ve)	Antihistamines only achieved limited effect.	Propranolol 20 mg 3 times a day: complete remission. Withdrawal of propranolol led to recurrence.

Legend *M* male, *F* female, *A* adrenaline, *AchH* acetyl(methyl)choline, *AM* Acetyl β methacholine chloride, *NA* noradrenaline, +ve positive, –ve negative, *DOS* diluted own sweat, *IDT-C* intradermal test in control(s), *IDT-P* intradermal test in patient, *ND* not described or not performed. Dosages of skin tests in Table 3

Apart from one case report, in which β-blockers induced cough, no side effects were described among cases listed in Tables 1 and 2, supporting their classification as a well-tolerated drug [19]. Studies have demonstrated an association between β-blocker use and increased anaphylaxis, which could be particularly relevant for patients with concomitant epinephrine auto-injector prescriptions for other allergic conditions [44]. The physiological effects of β-blockers could theoretically exacerbate the severity of anaphylaxis and reduce the effectiveness of epinephrine treatment by attenuating or abolishing its cardio stimulatory and bronchodilatory actions. Moreover, non-selective agents may induce unopposed alpha-adrenergic effects, potentially increasing the risk of bradycardia during epinephrine administration. Nevertheless, several clinical studies have concluded that there is insufficient evidence to support an association between β-blocker use and an increased frequency or severity of reactions [45–47].

**Table 2:** Characteristics of included case-series on usages of β-blockers in aquagenic induced pruritus, urticaria and aquadynia

Author, year	Type of study	Sex, Age	Diagnosis	Efficacy of earlier treatment attempts	Efficacy of β-blocking treatments
Cao, 2015 [18]	Case report	F, 36	3-year history of aquagenic pruritus	Various antihistamines were prescribed previously and were ineffective.	Propranolol 10 mg 2 times a day. At evaluation 1 month later: complete remission. The propranolol was converted to atenolol 25 mg once a day to circumvent the inconvenience of bidaily dosing. Symptoms remained adequately suppressed as long as she took the atenolol as prescribed. When missing a single dose, symptoms temporarily recurred that same day. Follow up for 16 months.
Hutten, 2025 [20]	Case series	F, 18	6-year history of aquagenic urticaria	Antihistamines were not tolerated.	Propranolol 20 mg in the morning and 10 mg in the evening. Complete resolution within 2 weeks of treatment. On days she forgot to take the propranolol, symptoms reappeared on the same day. Follow up for 2 years.
		F, 13	12-month history of progressively aquadynia and aquagenic urticaria	Triple therapy (several H1 blockers, 2 daily doses of 2 tablets; cimetidine, 2 daily doses of 400 mg; and montelukast, 1 daily dose of 10 mg) had only a subtle effect. Additional Omalizumab, 300 mg every 4 weeks, had a favorable effect with shortened episodes of erythema and dissolvement of pruritus and the burning sensation. After 2 years of omalizumab therapy, the treatment was discontinued on account of progressive side effects. Triple therapy was discontinued before the start with β-blockers.	Propranolol 10 mg 2 times a day: improvement of symptoms. Propranolol 20 mg 2 times a day: complete remission. In anticipated stressful situations, an additional 10-mg dose of propranolol was administered, yielding good results. Follow up for 6 months.
Nosbaum, 2011 [19]	Case series (*N* = 6)	3 F, 3 M, average age 41 (15–60 years)	10–30 years history of aquagenic pruritus (average duration of symptoms was 18 years)	Emollients (3 cases, ineffective), antihistamines (6 cases, 50% efficiency for 1 case, relapse after stopping), narrow-band UVB therapy (1 case, 100% efficiency, relapse after stopping), fluoxetine (2 cases, ineffective), clonidine (1 case, ineffective).	Propranolol 10 to 40 mg per day for 3 months, depending on their tolerance. Within 7 days, complete remission in 4 patients, 90% decrease of symptoms in 1 patient, no effect in 1 patient. No relapse occurred within 3 months of treatment, but after discontinuation of propranolol, clinical signs recurred in 5 patients. A cough was the only reported side effect induced by voluntarily doubling the dose of β-blockers (from 20 to 40 mg daily).
Rorie, 2016 [36]	Case report	F, 34	2-year history of aquagenic urticaria and aquagenic pruritus	No effect of simultaneous treatment with montelukast 10 mg daily, cetirizine 10 mg daily, loratadine 10 mg daily, ranitidine 150 mg 2 times a day, diphenhydramine 50 mg every night, and hydroxyzine 25 to 50 mg 30 min before bathing and frequently up to 3 times a day.	In addition to earlier treatment schedule: propranolol 30 mg daily gave no effect in urticaria, but mild improvement in the pruritus associated with water exposure. (She was then started on omalizumab 300 mg every 28 days and after 2 injections reported complete resolution of her aquagenic pruritus and urticaria.)
Shelley, 1998 [37]	Case series	F, 82	6-years history of aquadynia (Family history: sister polycythemia vera with associated aquagenic pruritus)	No effect of bicarbonate baths, hydroxyzine and other antihistamines.	Propranolol 60 mg per day provided partial relief in combination with atenolol, which she already had been using for two years prior to diagnosis; however, both the intensity and duration of pain sensations continued to increase. After two years, polycythemia was diagnosed. Phlebotomies had no effect on the aquadynia. Clonidine 0.1 mg 2 times a day (an imidazoline- and alpha 2 agonist) was subsequently prescribed, resulting in complete remission of pain.
		F, 49	12-year history of aquadynia	Some relief by taking ibuprofen 30 min before bathing and using biofeedback techniques, but none from various antihistamines.	She was taking propranolol 10 mg 2 times a day because of mitral valve prolapse. Long-acting propranolol 80 mg once a day resulted in substantial improvement. After 3.5 years, propranolol was discontinued without recurrence of pain during one year of follow-up.
Thomsen, 1990 [38]	Experience of author	ND	“Several cases” of idiopathic aquagenic pruritus not associated with polycythemia vera	ND	Propranolol 10 mg given administered 20 to 30 min prior to bathing markedly reduced pruritus.

*M* male, *F* female, *ND* not described

## Areas of uncertainty

### Cholinergic Urticaria and Adrenergic Urticaria

Regarding the pathophysiology of cholinergic urticaria, acetylcholine and cholinergic receptors at the neuroglandular junction of the sweat glands appear to be involved, as demonstrated in some but not all patients [48, 49]. For cholinergic urticaria, several distinct clinical features can be described; cholinergic urticaria with acquired anhidrosis or hypohidrosis versus without; solitary pruritus/urticaria versus pruritus/urticaria accompanied with a painful sensation; and follicular wheals versus non-follicular wheals [48]. Neither intradermal tests (IDT) nor in vitro tests are reliable as universal diagnostic tools due to their limited sensitivity (IDTs with cholinergic agents 50% [49, 50]; IDT with autologous sweat 33,3–64.7% [49, 51]; IDT with autologous serum 38,9–53.3% [49, 51]; and sweat induced histamine release from basophils in vitro 58.8% [49]. Furthermore, IDT cholinergic agents are not always reproducible in the same patient [50]. It is conceivable that a subset of the patients identified as cholinergic urticaria with false-negative test results for cholinergic urticaria may in fact represent true negatives, as their conditions should have been diagnosed as adrenergic urticaria since IDT with noradrenaline and adrenaline are rarely performed [52]. Although sensitivity rates are low, these tests may still be of additional value, as positive outcomes can reflect specific pathophysiological traits such as hypohidrosis, sweat hypersensitivity, and heightened cholinergic sensitivity. In cholinergic urticaria patients with positive IDTs to acetylcholine, skin lesions tend to be more severe and longer-lasting compared with those in patients with negative IDTs [48, 52–55].

In patients with adrenergic urticaria, β-adrenoreceptor-mediated responses may play an important role. A proposed mechanism involves sympathetic hyperactivity, resulting in the release of both epinephrine and norepinephrine. It remains unclear whether, in this subtype, the number of adrenergic receptors on mast cells is increased, the activation threshold is lowered, or whether noradrenaline exerts its effects in conjunction with an antigen [24]. Epinephrine induces vasoconstriction, which manifests as the blanched halo of the adrenergic urticaria lesion, whereas norepinephrine release triggers cutaneous mast cell degranulation, resulting in erythema, vasodilation, and oedema that constitute the erythematous center. IDTs with noradrenaline or adrenaline, but not with acetylcholine, are capable of reproducing the elementary lesions (Table 1). To avoid excessive vasoconstriction, highly diluted solutions must be used (Table 3). Several studies reported positive test results in patients with adrenergic urticaria, in contrast to negative responses observed in healthy controls or patients with cholinergic urticaria (Table 1). Some authors further noted that injection sites may present not with erythema, but with small wheals surrounded by a white halo as satellite lesions [40]. Further research should clarify the reproducibility, potential diagnostic value of IDTs with noradrenaline, adrenaline, and cholinergic agents in patients stating emotional or physical stress as luxating or exacerbating factors for urticaria. Since adrenergic urticaria can easily be misdiagnosed as cholinergic urticaria due to overlapping symptoms and triggering, further research should clarify if patients previously classified as cholinergic urticaria without positive IDT to acetylcholine may in fact represent patients with adrenergic urticaria. Furthermore, it would be of interest to determine whether the diagnostic and prognostic value of IDTs with cholinergic agents differs between subgroups with distinct clinical features of cholinergic urticaria [48].

**Table 3:** Dosages used for intradermal tests in the studies described in Table 1

Substance	Range of concentrations used in literature with positive results in patients	Patient(s)	Negative controls
Acetylcholine	10.000 ng/mL = 10 µg/mL = 0,01 mg/mL = 1:100.000	*N* = 1 [39]	
	100.000 ng/mL = 100 µg/mL = 0,1 mg/mL = 1:10.000	*N* = 1 [39], *N* [40	*N* = 1 [40]
	1.000.000 ng/mL = 1000 µg/mL = 1 mg/mL = 1:1000	*N* = 1 [39], *N* [40	*N* = 1 [40]
Methacholine chloride	0,1 mg in 0,1 mL saline	*N* = 1 [35]	
Adrenaline / epinephrine	500 ng/mL (= 10 ng in 0,02 ml saline)	*N* = 1 [22], *N* = 2 [24], *N* = 1 [40]	N = > 1 [24]
	750 ng/mL (= 15 ng in 0,02 ml saline)	*N* = 2 [14]	*N* = 7 [14]
	100.000 ng/mL = 100 µg/mL = 0,1 mg/mL = 1:10.000	*N* = 1 [31]	
	1.000.000 ng/mL = 1000 µg/mL = 1 mg/mL = 1:1000	*N* = 1 [29], *N* = 1 [31]	*N* = 5 [29]
Noradrenaline	150 ng/mL (= 3 ng in 0,02 ml saline)	*N* = 2 [14]	*N* = 7 [14]
	250 ng/mL (= 5 ng in 0,02 ml saline)	*N* = 2 [24], *N* = 1 [39]	N = > 1 [24]
	500 ng/mL (= 10 ng in 0,02 ml saline)	*N* = 1 [22], *N* = 2 [14], *N* = 1 [32]	*N* = 7 [14]
	1000 ng/mL = 1 µg/mL = 0,001 mg/mL = 1:1.000.000	*N* = 1 [42], *N* = 1 [39]	*N* = 10 [42]
	5000 ng/mL (= 100 ng in 0,02 ml saline)	*N* = 1 [40]	

If the dosage was not reported in the original article, it has not been included in this table

### The Aquagenic Spectrum

The pathophysiology of the aquagenic spectrum remains largely unclear. Over the years, various hypotheses have been proposed, including the involvement of water-soluble antigens [56, 57], exposure to alterations in osmolality [58], inappropriate activation of the autonomic nervous system [59–63], aberrant acetylcholine release [18], small fiber neuropathy involving C-fibres [18], sodium channel defects [18], and cutaneous fibrinolytic activity [64–66]. The role of histamine remains questionable, as antihistamines fail to induce (complete) remission in a substantial proportion of patients [58, 67]. To our knowledge, no randomized controlled trials have been conducted to elucidate the pathophysiology of the aquagenic spectrum or to evaluate the efficacy of antihistamines, omalizumab, parasympatholytics, or β-blockers [58].

### Β-Blockers

The mechanism of action of β-blockers in the described subset of chronic inducible urticaria remains poorly understood. It has been proposed that propranolol may inhibit mast cell degranulation by blocking β-2 receptors on the cell surface or may act centrally by reducing sympathetic drive and subsequent adrenergic receptor activation. Elucidating the mechanisms underlying β-blocker treatment could provide valuable insights into the pathophysiology of these chronic inducible urticaria subtypes. In one reported case of adrenergic urticaria with positive IDTs to noradrenaline, the urticarial response was abolished when the test was repeated after administration of oral propranolol 10 mg [42]. In two additional cases with adrenergic urticaria with aquagenic pruritus, local intracutaneous administration of propranolol and tolazoline suppressed IDT-induced urticaria and local pruritus, respectively, whereas local intracutaneous atropine did not inhibit this response in either patient (Table 4) [24]. Further research is warranted to assess the potential predictive value of repeated skin testing before and after single-dose propranolol treatment.

**Table 4:** Dosages used for local administration potentially interacting with intradermal tests

Substance			Solutions			*Patient(s)*
Atropine			0,05 mg (2 ml)			*N* = 2 [24]
Propranolol hydrochloride			0,1 mg			*N* = 2 [24]
Tolazoline hydrochloride			1 mg			*N* = 1 [24]

Given their high clinical impact, potential efficacy, low cost, broad availability, and favorable tolerability, we suggest that off-label use of low-dose β-blockers may be considered as a treatment option for adrenergic urticaria, cholinergic urticaria and the aquagenic spectrum [20]. Β-blockers may also have a role in chronic spontaneous urticaria and patients with ”Bier Anemic Spots, Cyanosis with Urticaria-Like Eruption“ (BASCULE) syndrome (Table 5) [68]. Further research is needed to elucidate the influence of interindividual pharmacokinetic and pharmacodynamic variability, as well as differences in tissue distribution and metabolite profiles on optimal dosing strategies, and to determine whether dose requirements consistently differ between chronic inducible urticaria subtypes.

**Table 5:** Usage of β-blockers in chronic spontaneous urticaria, a combination of subtypes of chronic inducible urticaria and the BASCULE syndrome

Author, year	Type of study	Sex, Age		Diagnosis	Efficacy of earlier treatment attempts				Efficacy of β-blocking treatments
Hutten, 2025 [41]	Case series	F, 19		5-year history of therapy refractory chronic inducible urticaria. Triggers included physical activity, vibrations, showering, and walking through the rain. Stress lowered the threshold for urticaria onset.	Fexofenadine, famotidine, montelukast, omalizumab, and cyclosporine all failed to obtain any disease control.				Propranolol 40 mg 2 times a day substantially improved symptoms, and the dosage of cyclosporine could be tapered.
		F, 17		8-month history of therapy refractory chronic spontaneous urticaria with stress as an obvious exacerbating factor but not an inducible factor.	Fexofenadine, famotidine, montelukast and omalizumab failed to obtain adequate disease control.				Propranolol 20 mg 2 times a day led to a 50% reduction in urticaria and pruritus within the first week of treatment. No dose escalation was performed at that time.
Reinhart, 2023 [68]	Case series (*N* = 3)	ND		Bier anemic spots, cyanosis with urticaria-like eruption (BASCULE) syndrome					Short-acting propranolol 10 mg 2 or 3 times a day in three patients whose prescription had been directed primarily toward autonomic dysfunction. Complete remission in 1 patient, 50% decrease of symptoms in 1 patient, decreased pain in showers in 1 patient.

Legend *F* female, *ND* not described

In our experience, clinical responses to β-blockers may occur rapidly, with effects typically observed within the first three days after initiation at a given dosage. As an initial step, we therefore typically start with propranolol 10 mg twice daily and instruct patients to increase the dose in 10 mg increments every three days until symptoms remit or a dose of 40 mg twice daily is reached. If symptoms are not fully controlled at this level, further up-titration can be considered. Given the off-label nature of this therapy, treatment within an *N* = 1 protocol - combined with the use of a patient diary, patient-reported outcome measures (PROMs), or repeated provocation tests during therapy - may be considered to objectively assess effectiveness. Future studies should evaluate how drug dosing regimens can improve clinical outcomes.

## Guidelines from Professional Societies

Both the international GA²LEN/UCAREs/ACAREs/EDF/APAAACI/ADD/BSACI/GA2CI

guideline for the definition, classification, diagnosis, and management of urticaria of 2026 and the AAAAI/ACAAAI joint task force guideline of 2014 do not report adrenergic urticaria nor aquadynia or aquagenic pruritus as specific subtypes of chronic inducible urticaria [7, 15]. Neither the BASCULE syndrome is described. The use of IDTs with cholinergic agents is only mentioned in the American guideline [15]. In diagnostic and treatment algorithms for chronic inducible urticaria, the primary recommendation is trigger avoidance; however, this is often difficult for patients to achieve and has a substantial impact on quality of life [69]. The recommended first-line pharmacological therapy is a second-generation antihistamine, which in most cases does not provide complete protection against trigger-induced whealing in these specific cases of inducible urticaria. The only additional option advocated is dose escalation. No licensed therapies beyond this are currently available for chronic inducible urticaria. Off-label omalizumab has been proposed; however, retrospective analyses indicate that its onset of effect is slower (early responders: 4·3% vs. 95·7%) and its efficacy lower (response rate: 68·2% vs. 93·5%) in patients with chronic inducible urticaria compared with those with chronic spontaneous urticaria [11]. Neither guideline addresses the possibility of off-label treatment with β-blockers.

## Conclusion

Chronic inducible urticaria can be challenging to treat; however, case series on β-blockers suggest potential beneficial effects and indicate similarities across the subgroups of cholinergic urticaria, adrenergic urticaria and the aquagenic induced disease continuum. Given their potential efficacy, low cost, broad availability, and favorable tolerability, we propose that off-label use of β-blockers may be considered an early treatment option for these conditions [20]. While trigger avoidance is commonly recommended, we believe it substantially impairs quality of life and may often be unnecessary in patients with adrenergic urticaria, cholinergic urticaria, and within the aquagenic continuum. Our review highlights several additional unmet needs in specific subgroups of chronic inducible urticaria. Further studies are required to elucidate the pathogenesis underlying the occurrence of different urticaria subtypes. The inclusion of adrenergic urticaria, adrenergic pruritus, aquagenic pruritus, and aquadynia in clinical guidelines, as well as the BASCULE syndrome as a differential diagnosis, and IDTs with standardized concentrations, may help prevent misclassification of these subtypes. Moreover, further research should assess whether intradermal testing can aid in predicting treatment outcomes in these patient groups.

### Search Strategy and Selection Criteria

We searched PubMed from database inception to July 2025, using the terms “Beta-blockers AND Urticaria”, “Propranolol AND Urticaria”, “Adrenergic Beta-Antagonists AND Urticaria”, “propranolol AND aquagenic”, “Adrenergic Beta-Antagonists AND aquagenic”, and “Adrenergic urticaria”. We also reviewed current guidelines for the management of urticaria and examined reference lists of the retrieved articles for additional sources. Language was restricted to Dutch, German, and English. Only studies reporting results in human were considered relevant.

## Data Availability

Data sharing is not applicable to this article as no new data were created or analyzed in this study.

## Abbreviations

IDT: Intradermal tests
BASCULE: Bier anemic spots, cyanosis with urticaria-like eruption
